# Current Status of Raf Kinase Inhibitor Protein (RKIP) in Lung Cancer: Behind RTK Signaling

**DOI:** 10.3390/cells8050442

**Published:** 2019-05-10

**Authors:** Ana Raquel-Cunha, Diana Cardoso-Carneiro, Rui M. Reis, Olga Martinho

**Affiliations:** 1 Life and Health Sciences Research Institute (ICVS), School of Medicine, University of Minho, Campus de Gualtar, 4710-057 Braga, Portugal; a.raquelfcunha@gmail.com (A.R.-C.); diana.ccarneiro@gmail.com (D.C.-C.); rreis@med.uminho.pt (R.M.R.); 2 ICVS/3Bs-PT Government Associate Laboratory, 4710-057 Braga/4805-017 Guimarães, Portugal; 3 Molecular Oncology Research Center, Barretos Cancer Hospital, Barretos, São Paulo 14784 400, Brazil

**Keywords:** lung cancer, EGFR, RKIP signaling, prognosis, cancer therapy

## Abstract

Lung cancer is the most deadly neoplasm with the highest incidence in both genders, with non-small cell lung cancer (NSCLC) being the most frequent subtype. Somatic mutations within the tyrosine kinase domain of the epidermal growth factor receptor (EGFR) gene are key drivers of NSCLC progression, with EGFR inhibitors being particularly beneficial for patients carrying the so-called “EGFR-sensitizing mutations”. However, patients eventually acquire resistance to these EGFR inhibitors, and a better knowledge of other driven and targetable proteins will allow the design of increasingly accurate drugs against patients’ specific molecular aberrations. Raf kinase inhibitory protein (RKIP) is an important modulator of relevant intracellular signaling pathways, including those controlled by EGFR, such as MAPK. It has been reported that it has metastasis suppressor activity and a prognostic role in several solid tumors, including lung cancer. In the present review, the potential use of RKIP in the clinic as a prognostic biomarker and predictor of therapy response in lung cancer is addressed.

## 1. Introduction

### 1.1. Lung Cancer

Of the several existing types of cancer, lung cancer is the most common and fatal for both male and females [1]. It is estimated that 1.8 million new cases and 1.6 million deaths occur every year [2]. Lung cancer is usually divided into two main categories: small cell lung cancer (SCLC) and non-small cell lung cancer (NSCLC). The latter is further divided into three major types, squamous cell carcinoma (SCC), adenocarcinoma (AC), and large cell carcinoma [3]. Lung AC is the most common type of lung cancer and has a poor survival rate. Only 10% of cases have a life expectancy of five years and those that are not treated and, on average live only four months, which is usually a result of late detection and a lack of treatments for the advanced stages [4,5].

Tobacco is the main risk factor for lung cancer, and although the number of new cases registered is high, it is believed that in Western countries the epidemiological peak of lung cancer has already occurred, as the incidence and death rates are decreasing [1,3,6,7]. There is a significant difference between lung-cancer patients who have never smoked and those who have a smoking history. The differences are found in the incidence, phenotype, genotype of tumors and overall patient survival, providing evidence that these cancers arise through different molecular mechanisms [3]. In fact, ACs in patients that have never smoked frequently harbor mutations in the epidermal growth factor receptor (*EGFR*) gene. On the other hand, smoking patients commonly harbor mutations in the *KRAS* and *TP53* genes [8,9,10]. This is possibly explained by exposure to highly carcinogenic agents like tobacco smoke, which might specifically induce mitogen-activated protein kinase (MAPK) signaling pathways through mutations in *KRAS*. For patients who have never smoked, the yet unidentified carcinogens might selectively target the EGFR pathway by inducing mutations in the gene [3].

EGFR is a receptor tyrosine kinase (RTK), which is a highly conserved family of transmembrane receptors for extracellular signaling molecules, such as growth factors and hormones [11]. Upon activation of these receptors, several major signaling pathways, such as MAPK, PI3K/AKT, and JAK/STAT, can be activated, ultimately leading to alteration of gene expression and protein functions responsible for critical cellular processes like cell survival, proliferation, and differentiation [11,12,13]. EGFR is frequently upregulated in NSCLC, and is a biomarker that is prognostic for a poor clinical outcome [14]. Increased receptor activity can be due to either overexpression of activating ligands (e.g., EGF), mislocalization of the receptor [15], or, more frequently, oncogenic driver mutations, such as deletion of exon 19 (45% of *EGFR* mutations), the point mutation L858R in exon 21 (40% of EGFR mutations), and in-frame insertions within exon 20 (5–10% of *EGFR* mutations) [16]. 

Understanding of the disease at the molecular level has allowed identification of specific molecular targets for which drugs can be identified or engineered to inhibit [17,18]. This approach has proven to be remarkably successful, as there are now several molecular targets and FDA-approved drugs for advanced-stage NSCLC [19,20,21,22]. Table 1 summarizes the most common targets and their corresponding approved drugs to treat NSCLC cancer patients, which will be further explored below.

The first generation of EGFR tyrosine kinase inhibitors (EGFR-TKIs) were gefitinib and erlotinib, which bind reversely to the ATP binding site of the receptor, and hence the presence of EGFR-activating mutations can predict a stronger clinical response [23]; patients with these mutations have longer progression-free survival (PFS) when compared with those treated with conventional chemotherapy [24,25,26]. Although promising, tumors treated with first-generation EGFR-TKIs inevitably develop resistance with time, mainly due to acquisition of secondary mutations, such as T790M in exon 20 [27], or due to reverting the “EGFR-addicted” stage to other RTKs, such as MET and HER3 [28,29].

In an attempt to circumvent the development of resistance, a second generation of TKIs was developed, such as afatinib and dacomitinib, which are able to irreversibly inhibit the tyrosine kinase activity of EGFR, HER2, and HER4 [30,31,32]. Osimertinib is, for now, the only third-generation inhibitor that has FDA approval, and shows significant improvement of clinical endpoints, including PFS [33,34,35]. Initially osimertinib was approved for the treatment of metastatic EGFR T790M mutant NSCLC, after the patient had progressed on first- or second-generation EGFR TKIs, however, it is now also used as a first-line treatment for EGFR mutant lung cancer patients [34,35].

For *EGFR* wild type patients, *KRAS* mutations are the most incident, occurring mainly at exon 2 codon 12 and more rarely at exon 3 codon 61, causing loss of *KRAS* GTPase, leading to the stimulation of effector proteins independently of the upstream receptor activity [36]. Unfortunately, an efficient targeted therapy against it has not yet been developed, and so patients harboring KRAS mutations are instead redirected for chemotherapy [37,38,39]. Another example of MAPK pathway alterations, which are mutually exclusive with *EGFR*, is *BRAF* mutations [40], for which there are specific inhibitors available (Table 1) [41].

Although to a lesser extent, NSCLC tumors can be characterized by alterations in other important molecular targets, such as *ALK* translocations [42] or *MET* mutations [28,29], for which the drug crizotinib has been used (reviewed in [43]). 

In spite of the above-cited success, major challenges remain concerning treatment of lung cancer, such as targeting the “undruggable” oncogenic drivers, such as mutant *KRAS*, or understanding why responses to molecular targeted therapies in NSCLC are almost always incomplete and transient. With continued research, there is great promise for continued advances in the biomarker-driven treatment of patients with NSCLC.

### 1.2. Raf Kinase Inhibitory Protein (RKIP)

Raf kinase inhibitor protein (RKIP), also known as PEBP1 (phosphatidylethanolamine-binding protein 1), is a 23 kDa protein originally purified from the bovine brain, and is mainly present in the cytoplasm, but also at the plasma membrane and in the nucleus of a great variety of tissues [44,45,46,47]. Functionally, this ubiquitous protein was firstly described as being an interactive partner of Raf-1, a kinase of the MAPK cascade, named because it inhibits Raf-1 [48,49]. Later on, RKIP was also described as an important modulator of other cellular pathways, such as the G-protein-coupled receptor (GPCR) kinase pathway, the nuclear factor kappa b (NF-κB) cell survival pathway, and the GSK3 cascade [49,50]. RKIP is by itself a phosphoprotein, having a phosphorylation site at serine 153 that has been shown to be a target of protein kinase C (PKC). PKC-mediated phosphorylation of RKIP decreases its affinity for Raf-1 and increases its affinity for GRK2, reversing its MAPK inhibitory function [51,52].

RKIP is widely expressed in normal human tissues, being recognized as having an important role in various physiologic processes [53,54]. In cancer, RKIP is considered to be a tumor suppressor, with its lost or reduced expression being associated with malignancy and poor prognosis in several tumor types, as described by our [47,55,56,57,58,59] and other [60,61,62] groups. The first association between RKIP and cancer was established in prostate tumors, in which cellular RKIP expression levels were below average and were even lower in metastatic tumors [63]. On the other hand, Fu et al. also demonstrated that when RKIP expression was reestablished in metastatic cells, their invasion capacity was inhibited, but the growth of the primary tumor was not affected [63]. This suggested that RKIP cannot have a central role in the primary tumor, but instead has great importance as a metastasis suppressor [63,64]. It is now known that RKIP is a multifunctional protein in carcinogenesis, and through the modulation of different intracellular signaling pathways, it is able to control cellular growth [65,66], motility [67,68], epithelial–mesenchymal transition (EMT) [69] and invasion [70]. Importantly, it was also established that RKIP downregulation leads to a dramatic inhibition of apoptosis and development of chemoresistance to conventional cytotoxic drugs in tumor cells [62,71].

Despite the increased importance of RKIP as a metastatic and prognostic marker in human cancer, the mechanisms behind its downregulation remain elusive [72]. Some evidence suggests that it might be downregulated through transcriptional or post-transcriptional mechanisms, such as promotor methylation [73,74], transcription inhibition through SNAIL [75], and BACH1 [76], or by microRNAs such as miR-224 and miR-27a [77,78].

Deletion or downregulation of RKIP has also been described in other pathologies, such as Alzheimer’s disease, diabetic nephropathy, sperm decapitation, heart failure [53,54,79,80,81], or lung-associated pathologies [82,83,84]. Specifically, RKIP has been implicated in asthma progression due to its interaction with epithelial 15-lipoxygenase 1 (15LO1), a molecule that was shown to be overexpressed in this disease. Low levels of colocalization of Raf-1 and RKIP, but high levels of colocalization of 15LO1 with RKIP, were observed in human asthmatic tissues when compared with normal tissue [82]. The interaction between 15LO1 and RKIP was described as a regulator of ferroptosis, a form of programmed cell death that is pathogenic to several acute and chronic diseases [83]. Hence, the RKIP/15LO complex has great potential to be a new target for drug discovery against such diseases.

Additionally, it has been shown in human bronchial epithelial cells that upon long-term exposure to cigarette smoke, acetylcholine (Ach) levels are raised, promoting the phosphorylation of RKIP, ERK1/2 and β2-adrenergic receptors by enhancing PI3/PKC/RKIP/Raf-ERK1/2 pathway activation [84]. Hence, besides the desensitization of heterologous β2-adrenergic receptors, increasing levels of molecules such as phospho-RKIP might also aggravate the inflammatory and oxidative stress in the airways of smokers, promoting bronchial epithelial cell activation. These discoveries might be particularly important for the treatment of patients with chronic obstructive pulmonary disease (COPD), as inflammation and oxidative/nitrosative stress are considered of primary pathogenic importance [84].

Overall, the previous reviewed data, show that RKIP is an important modulator of relevant intracellular signaling pathways (including MAPK, which is controlled by EGFR), is both a driver and predictor of therapy response in lung cancer (Table 1), and has an important role in normal lung homeostasis. This has led us to hypothesize that this metastasis suppressor protein could be of upmost importance in lung tumors. Its expression levels, prognostic value, and biological role in lung cancer will be reviewed in the following sections.

## 2. RKIP and Lung Cancer: Literature Review

### 2.1. Expression and Prognostic Value 

Regarding RKIP expression and its clinical significance in lung cancer, we have found that the studies available are still scarce and inconclusive, and are not concordant among them (summarized in Table 2) [85,86,87,88,89,90].

In general, RKIP mRNA was detected in 41.9% and 47.7% of NSCLC patients [85,86], while RKIP protein positivity has been described by immunohistochemistry (IHC) in among 49.1% to 64.5% of NSCLC patients [87,88,89]. This was independent of the histological type, as all the studies that compared AC with SCC found no statistically significant differences among them, both at the mRNA and protein levels (Table 2) [86,87,88]. Additionally, there is one study that assessed the expression levels of the inactive form of RKIP, with phosphorylation at serine 153 (pRKIP) [90]. The study is unclear because the authors do not state the percentage of positive cases, but from their survival curves we were able to estimate that the minority of patients expressed pRKIP, corresponding to 37.6% (140/372) of the samples (Table 2) [90]. Overall, the studies are not comparable due to the different techniques (RT-PCR vs. QPCR), or antibodies and methodologies (IHC), used. The highest discrepancies were found in protein studies (Table 2) as the variability inherent to IHC studies is well-known. Furthermore, the median percentage of RKIP positive cases is higher in IHC studies than in mRNA ones, which is probably also due to the variability and lower specificity of IHC (Table 2).

RKIP is a well-established metastasis suppressor [63,91,92]. It is described in several tumor types as underexpressed in primary tumors when compared to normal tissues, and significantly decreased or even absent in metastases [60]. In lung cancer, Zhu C et al. found a significant reduction of RKIP mRNA expression levels in tumor tissues when compared to the surrounding normal tissues, which showed 76.7% (66/86) RKIP positivity (Table 2) [85]. In accordance, even without specifying the percentage of positive samples, Wang Q et al. described a similar difference between normal and tumor samples by QPCR [86].

At the protein level, Huerta-Yepez S et al. compared the expression levels of RKIP between lung tumor and normal tissues in a large series of samples (671 lung tissues) by analyzing both RKIP and pRKIP expression through an IHC approach [90]. The authors did not find differences in total RKIP expression levels between normal epithelium, primary NSCLC, or metastatic lesions, but instead described a slight statistically significant decrease in pRKIP expression in metastatic, compared to nonmalignant, lesions [90]. Consistently, they also showed that lower levels of pRKIP are correlated with poor outcome, however, contradictorily, that was not concordant with their own findings, which showed that a higher pRKIP expression level is associated with aggressiveness markers, such as age and presence of lymph node metastases [90]. Looking deeply into the data, the results are unexpected, because, by concept, RKIP phosphorylation at serine 153 dissociates RKIP from Raf-1, reversing its inhibitory function [51]. Therefore, it is expected that expression of pRKIP should result in a poor outcome. In fact, for other tumors, such as multiple myeloma and stage II colon cancer, pRKIP may contribute positively to overall cell survival and drug resistance, and hence, tumor aggressiveness [93,94]. However, other studies in melanoma and breast cancer, such as the study by Huerta-Yepez S et al. [90], have also shown that low levels of pRKIP could predict poor survival in comparison with relatively higher expression [95,96]. Thus, the clinical differences of pRKIP in different tumors are worth further study.

Recently, using a different antibody for total RKIP, Wang A et al. observed negative or weak staining of RKIP in the majority of lung tumor tissues, compared with the intense staining of noncancerous tissues, as expected [89]. Furthermore, even though they did not analyze RKIP expression in metastatic tissues as in the Huerta-Yepez S et al. study [90], the authors found a statistically significant association between low total RKIP protein expression levels and higher TNM stage, and presence of lymph node and distant metastases [89], a result that is concordant with the remaining studies available, either at the mRNA [85,86] or protein level [87,88] (Table 2).

Regarding the predictive role of RKIP in the prognosis of lung cancer patients (Table 2), the results are also ambiguous. As cited above, Huerta-Yepez S et al. [90] found that low levels of pRKIP were an independent poor prognostic marker, while total levels of RKIP had no predictive value in their cohort of patients [90]. However, a more recent study showed that a decrease in the total levels of RKIP expression constitutes an independent poor prognostic marker in NSCLC patients, as assessed by IHC in primary tumors [88]. Interestingly, the authors also showed that the RKIP expression level was generally lower in radioresistant NSCLC tissues, pointing out its putative role in radiotherapy response modulation [88]. The difference among the two studies is most likely because the first study used tissue microarrays (TMAs), which we showed previously was not the best way to study RKIP expression because it requires the largest representative sample possible as loss of RKIP expression in primary tumors is essentially focal [47,55,56,57,59,97]. Technical problems explaining the difference are unlikely because Huerta-Yepez S et al. [90] used the same antibody (from Millipore, Upstate Biotechnology) that we and other authors have used without problems [47,55,56,57,59,97]

Importantly, two distinct papers showed that RKIP expression levels are associated with the expression of other cancer-related proteins in lung tumor tissues, such as positive expression of E-Cadherin [85] and negative expression of phosphorylated STAT3 [89]. The associations between RKIP, E-cadherin, and STAT3 are not novel, and are also well described in other tumor types (as reviewed in [62]), emphasizing the biological importance of studying RKIP in lung cancer, as will be reviewed in the next section.

Although many questions remain regarding the best method to reliably detect RKIP expression levels in lung cancer, most studies agree on the clear association between low expression of RKIP and higher TNM stage or presence of lymph node metastases (Table 2). More studies are still needed to validate its prognostic value, both in its active or inactive (pRKP) form.

### 2.2. RKIP Biological Role: A Modulator of Cell Signaling

**Figure 1:**
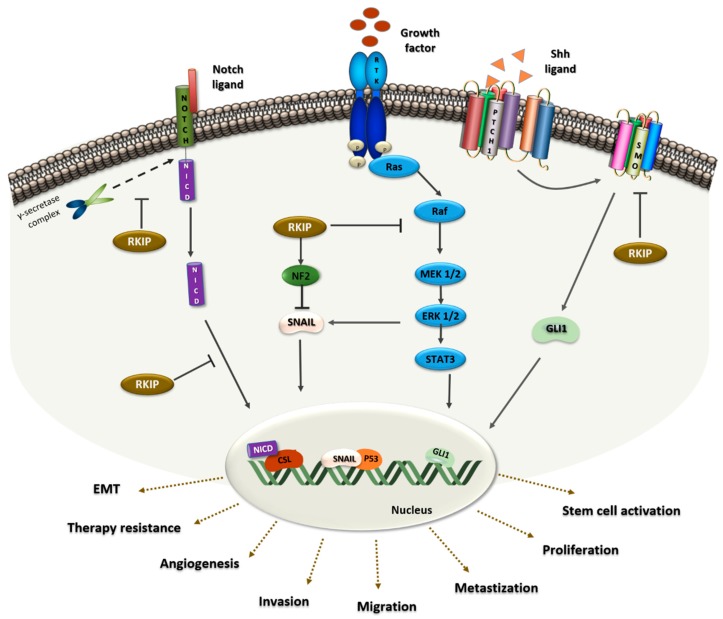
RKIP protein as a signaling modulator in lung cancer. On the left, RKIP binds to the Notch Intracellular Domain (NICD) preventing the proteolytic cleavage by the γ-secretase complex. Furthermore, in the Notch1 pathway, RKIP inhibits the translocation of NICD to the nucleus, which would then activate the translocation of EMT-related genes, ultimately promoting cell invasion and metastasis. In the middle, RKIP binds to Raf, preventing the phosphorylation of MEK by Raf and consequently, Raf/MEK/ERK/STAT3 signaling is inhibited. This will enhance events such as angiogenesis, proliferation and metastization. Additionally, RKIP blocks Snail through MAPK inhibition and NF2 stabilization. In the nucleus, SNAIL acts as a p53 suppressor and upon this EMT related-processes will occur. On the right, RKIP act as an inhibitor of the Shh signaling pathway. RKIP binds to the SMO receptor, keeping it inactive and preventing Gli1 transcription, and promoting therapy resistance and stem cell activation.

The first evidence showing RKIP as a modulator of cell signaling in lung cancer came from in vitro experiments using a *KRAS* adenocarcinoma mutated cell line (A549) [89]. The authors demonstrated, by lentiviral overexpression, that RKIP decreases the levels of IL-6 dependent ERK and STAT3 phosphorylation (Figure 1) and, consequently, the cells migratory capacity [89]

Furthermore, it was demonstrated that RKIP acts as a physiological inhibitor of NOTCH1, a major player in EMT and metastases [98]. Using H1299 cells, transfected to overexpress RKIP, the authors demonstrated that RKIP directly interacts with the full-length of NOTCH1, preventing its proteolytic cleavage and NICD release (Figure 1), decreasing EMT markers like Vimentin, N-cadherin and Snail. As a consequence, the migratory and invasive capacity of the cells also decreased, a phenotype that was reverted *in vivo* by *RKIP* knockdown in A549 cells [98].

Signaling axes involving RKIP and microRNAs were also described in NSCLC as important modulators of EMT and metastasis [77,99]. Using the A549 cell line, it was found that, by downregulation of RKIP, miR-27a increases Vimentin expression, as well as cell invasion capacity, and decreases E-cadherin levels [77]. Furthermore, it was demonstrated that dysregulation of the miR-150-FOXO4 axis promotes EMT through modulation of the NF-κB /SNAIL/YY1/RKIP loop [99]. In vitro assays showed that miR-150 downregulates FOXO4, resulting in increased levels of NF-κB and its targets, SNAIL and YY, which in turn, will lead to RKIP downregulation [99]. 

RKIP was also identified as a p53 modulator in malignant pleural mesothelioma (MPM), an asbestos-induced human lung cancer [100]. Using MPM and NSCLC cell lines treated with silica, an increase in ERK activation and a decrease in p53 expression levels promoted by RKIP depletion were observed. In this RKIP tumor-promoting context, MAPK signaling activation and neurofibromatosis 2 (NF2) protein inactivation triggers SNAIL expression that ultimately leads to p53 and E-cadherin inhibition (Figure 1) [100].

Furthermore, it was demonstrated that the expression levels of the signal transducer Smoothened (SMO) and Gli1, a zinc-finger transcription factor, are decreased in RKIP knockdown cells, pointing to RKIP as an inhibitor of Sonic Hedgehog (Shh) signaling [88]. Briefly, in the activated state of the pathway, the ligand Shh binds to a transmembrane protein receptor, Patched-1 (PTC1), which loses its catalytic inhibition of SMO (Figure 1). Consequently, active SMO will trigger the transcription of the Shh target gene Gli1, which acts as a transcriptional activator of numerous genes, regulating proliferation, differentiation, extracellular matrix interactions, and cancer stem cell (CSC) activation [101]. Mechanistically, RKIP binds to SMO keeping it inactive and preventing the transcription of Gli1 (Figure 1) [88].

Finally, as referred to before, RKIP was established as a metastasis suppressor for the first time in prostate cancer, where it was reported that low RKIP expression in primary tumors increases the probability of lung metastasis development [63], a finding that was further demonstrated for other tumors of different primary sites [102,103,104,105]. Beshir et al., by using a breast cancer orthotopic model injected with RKIP expressing cells, showed that tumors expressing RKIP formed less lung metastases [102]. Later on, in an attempt to understand the mechanism behind this event, Dattar et al. proposed that RKIP inhibits the occurrence of lung metastases through the regulation of the CCL5 protein and the reduction of macrophage lung infiltration [103]. Similarly, in the nasopharyngeal carcinomas, RKIP downregulation promotes invasion, metastasis and EMT by activating STAT3 signaling [104]. Using a different approach, it was recently shown in the hepatocellular carcinomas, that somatostatin octapeptide significantly reduced the occurrence of pulmonary metastases *in vivo* by increasing RKIP levels in the primary tumor [105].

### 2.3. RKIP Implications in Therapy Response

RKIP has been reported as an important molecular player in the modulation of tumor cells resistant to conventional therapies, however the mechanisms behind this remain largely unclear [106,107,108,109].

Concerning NSCLC, it has already been reported in vitro that A549 cells treated with the chemotherapeutic agents, adriamycin and 9NC, increased RKIP expression in a time and dose dependent manner. Activation of RKIP expression is, in the case of adriamycin, fully dependent on the p53 transcription factor, which is able to bind to RKIP’s promotor at two different binding sites [107].

Moreover, gemcitabine, another chemotherapeutic drug, also induces RKIP expression not only in the A549 cell line, but also in the CALU-1, CALU-6, H23, and HCC 827 cell lines [110]. The authors demonstrated that gemcitabine and sorafenib—an oral multikinase inhibitor that decreases the kinase activity of both C-RAF and BRAF—interact with each other, resulting in potent inhibition of cell proliferation and induction of apoptosis. In this synergistic interaction, Raf inhibition, a pharmacologic effect of sorafenib, is enhanced by gemcitabine as a consequence of its ability to induce RKIP expression [110]. NF-κB activation was suggested by the authors to be a possible mechanism for gemcitabine-mediated RKIP induction, a consequence of DNA damage induced by the same drug [110]. Later, Giovannetti et al. also studied the synergistic interaction between sorafenib and erlotinib, reporting that sorafenib slowed cell cycle progression and induced apoptosis, which was significantly increased with the combination of drugs [111]. Moreover, sorafenib-related reduction of AKT/ERK phosphorylation in erlotinib-resistant cells (A549 and H1975) was associated with significant RKIP upregulation, probably by NF-κB activation, a consequence of erlotinib’s EGFR inhibition [111].

RKIP was further implicated in the mechanism through which miR27a regulates cisplatin resistance in the A549 cell line [77]. Li et al. reported, both in vitro and *in vivo*, that miR27a appears to be increased in cisplatin-resistant A549 cells when compared with the parental A549 cell line, while RKIP, which they report as a direct target of miR27a, appears to be decreased. RKIP knockdown in the A549 cell line decreased the sensitivity to cisplatin, while ectopic expression of RKIP, in part, rescued miR27a-mediated resistance to cisplatin [77]. Importantly, the authors were also able to demonstrate an association between miR27a and RKIP expression with chemotherapeutic resistance using clinical tumor tissue samples collected from patients with advanced lung adenocarcinoma [77]. 

Similarly, Xie et al., through the analysis of RKIP expression in a series of human NSCLC tissues divided into radiosensitive and radioresistant, reported that RKIP expression levels were positively correlated with radiosensitivity [88]. Accordingly, the authors demonstrated in vitro that both the A549 and SK-MES-1 cell lines, with RKIP knockdown, showed increased resistance to different degrees of radiotherapy as well as lower radiation-induced apoptosis [88]. The modulation of the Shh pathway, specifically its activation through RKIP depletion, was one of the mechanisms proposed to explain radioresistance. The authors demonstrated that, in RKIP knockdown cells, Gli1 overexpression increased the number of CSCs, somehow explaining the observed radioresistance *in vivo* [88].

## 3. RKIP and Lung Cancer: In Silico Analysis

Recently, Zaravinos A and colleagues, by analyzing RKIP mRNA expression across 37 different cancer types and using data from The Cancer Genome Atlas (TCGA) platform, showed that RKIP is downregulated compared to normal lung tissues, with lung adenocarcinoma being among the eight tumor types with the lowest RKIP expression levels [62]. Another study, using the same database, suggested that RKIP downregulation in cancer is not due to genetic or mutation events, but rather to transcriptional or post-transcriptional mechanisms [61]. Even so, breast cancers, gliomas, and NSCLCs seem to present the highest RKIP genetic heterogeneity among the 25 tumor types analyzed [61].

**Figure 2:**
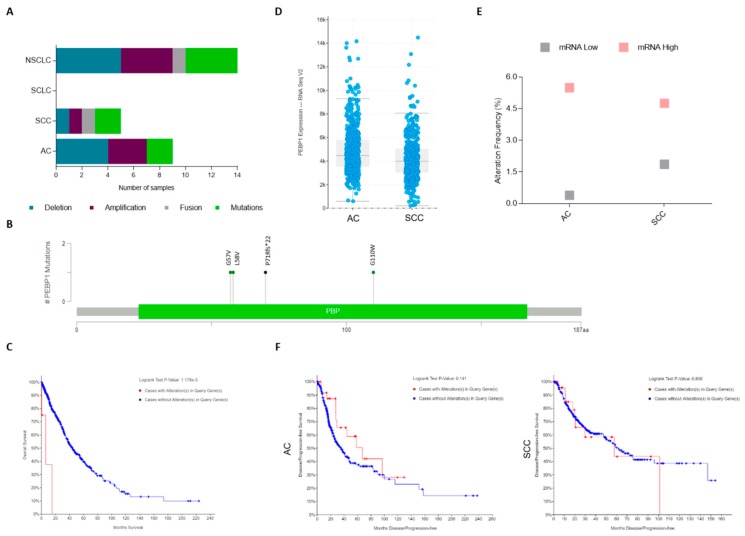
Lung cancer The Cancer Genome Atlas (TCGA) data for genomic alterations on the RKIP encoding gene (*PEBP1*). (**a**) Number of cases depicting RKIP genomic alterations in the different histological types (from an analysis of 17 different studies containing 4028 samples). (**b**) Scheme showing distribution of *PEBP1* mutations in the entire RKIP protein. (**c**) Kaplan–Meier analysis of NSCLC patient’s overall survival (OS) in months distributed by the presence (red line: 6.18 months of median OS, from 6 patients) or absence (blue line: 43.91 months of median OS, from 948 patients) of RKIP gene alterations (*p* < 0.05). (**d**) RNA Seq V2 data, showing the mean of RKIP mRNA expression levels in AC (566 patients) and SCC (487 patients). (**e**) Percentage of cases depicting mRNA up and downregulation in the different NSCLC histological types (refers to a total of 1094 cases). (**f**) Kaplan–Meier analysis of NSCLC patient’s progression-free survival in months, distributed by the presence (red line, 31 cases for AC and 27 SCC) or absence (blue line, 475 cases for AC and 348 SCC) of RKIP mRNA alterations. All data is available at www.cbioportal.org. SCLC: Small Cell Lung Carcinoma; NSCLC: Non-Small Cell Lung Carcinoma; AC: Adenocarcinoma; SCC: Squamous cell Carcinoma.

Analyzing the TCGA data with regard to lung cancer [112,113,114,115,116,117,118,119,120] shows that there are 17 studies available at cBioPortal database (www.cbioportal.org) that account for more than 4028 samples. We found that RKIP molecular alterations are in fact a rare event (<0.5% of altered cases among the 4028), and they occur exclusively in NSCLC (Figure 2A and Table 3). In total, there are 14 cases that depict *PEBP1* gene alterations: three missense mutations, one frameshift deletion, one fusion with *HECTD4* gene, four cases with gene amplification, and five with homozygous deletion (Table 3). Remarkably, disregarding the case with gene fusion, all other mutated cases (4/5) have hotspot mutations in known lung cancer-related genes, while those alterations were found in only 44% (4/9) of the tumors with *PEBP1* copy number alterations (CNA) (Table 3). Regarding *RKIP* mutations, all were found in exons 2 and 3 at the phosphatidylethanolamine-binding domain of the protein (Figure 2B). Interestingly, even though it is a rare event, a significant association was found between the presence of genomic alterations in the *PEBP1* gene and a poor overall survival in NSCLC (Figure 2C); knowing this, it could be interesting to include mutational and CNA analysis in the studies aiming to explore the prognostic value of RKIP in the future.

Regarding mRNA expression, relative to RNA-seq data available from the TCGA PanCancer Atlas [112,113,114,115,116,117], we observed that SCC cases present lower mean levels of RKIP mRNA when compared to AC (Figure 2D). Specifically, categorizing the patients by RKIP mRNA up and downregulation (as defined by the cBioPortal settings), mRNA upregulation was found in around 4.7% of the total cases (51/1094), including both AC (5.49%, 28/510), and SCC (4.75%, 23/484), while data for the RKIP mRNA downregulation (1%, 11/1094) was higher in SCC (1.86%, 9/484) compared to AC (0.39%, 2/510) (Figure 2E). The remaining cases (94%, 1032/1094) are described in the database as having “no altered” mRNA expression (i.e., up or downregulated), and are considered positive with normal expression levels of RKIP mRNA. No statistical associations were found between RKIP mRNA expression and patient survival, still, patients with lung AC overexpressing RKIP have a double progression-free survival time when compared with the ones with no alteration in RKIP mRNA (67.18 vs. 35.58 months) (Figure 2F).

**Figure 3:**
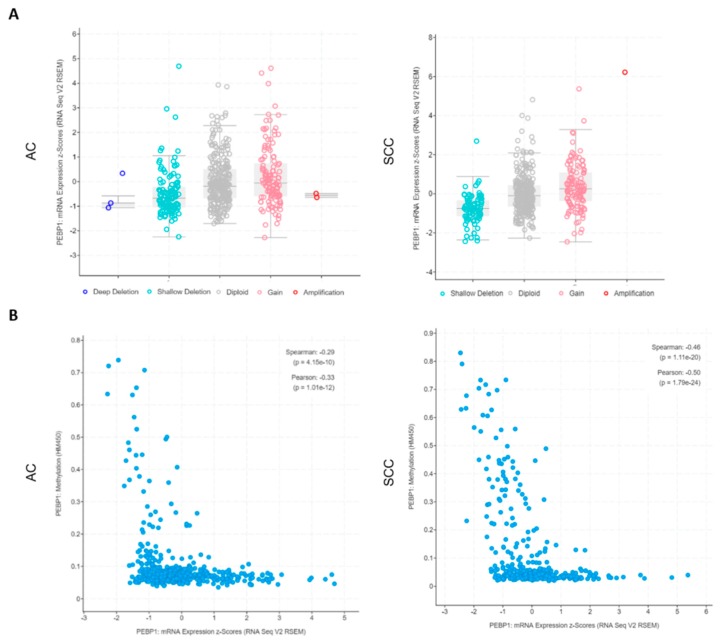
NSCLC TCGA data regarding RKIP encoding gene (*PEBP1*) alterations. (**a**) RKIP mRNA expression levels according to the copy number variations status (X axis) of the *PEBP1* gene (refers to 515 AC and 501 SCC). (**b**) Correlation between the levels of RKIP mRNA expression and methylation status of the *PEBP1* gene (refers to 515 AC and 501 SCC). All data is available at www.cbioportal.org. NSCLC: Non-Small Cell Lung Carcinoma; AC: Adenocarcinoma; SCC: Squamous cell Carcinoma.

Additionally, stratifying the patients by CNA, we can unequivocally observe that RKIP mRNA expression levels vary and are wholly associated with the copy numbers of the gene in both histological types (Figure 3A), with CNA strongly associated with the 12q chromosome status (Supplementary Figure S1). Additionally, we were able to confirm for AC samples (the only samples with methylation data available) that there is a good negative correlation (Pearson = −0.33, *p* = 1 × e^−12^) between low mRNA expression levels and high methylation status of the gene (Figure 3B). 

## 4. Conclusions

The development of therapies targeting RTKs, particularly EGFR, has been of great significance for lung cancer treatment. The role of RKIP as a prognostic marker in lung cancer is not yet clear, as this matter is still controversial in the literature. Nonetheless, the implication of RKIP in tumor-related signaling and ultimately in therapy resistance is clear. Understanding how RKIP is positioned in resistance responses, in this type of cancer, could be crucial for the reversion of this problem. This would enable a confident establishment of RKIP’s role in lung tumors, possibly potentiating its successful translation into the clinic.

## Figures and Tables

**Table 1:** Molecular targets in non-small cell lung cancer (NSCLC), their prevalence, patient characteristics and the targeted therapies applied (FDA approved). Adapted from [22].

Molecular Target	Prevalence (%)	Commonly Associated Patient Characteristics	Agent Used in Targeted Therapy
RAS	30	Former/current smokers	None
EGFR	10–18 (Caucasian) 40–55 (Asian)	East Asian, female, never smokers	Afatinib, Dacomitinib, Erlotinib, Geftinib, Necitumumab, Osimertinib
ALK	3–7	Young, never smokers	Alectinib, Ceritinib, Crizotinib
ROS	1–2	Young, never smokers	Brigatinib, Crizotinib,
RET	1–2	Never smokers	Vandetanib, Cabozantinib
BRAF	3–5	Former /current smokers	Dabrafenib
HER2	1–4	Female, never smokers	Afatinib, Transtuzumab
MET	11	Mutually exclusive with EGFR mutations	Crizotinib, Onartuzumab

**Table 2:** Raf kinase inhibitory protein (RKIP) expression and its clinical impact in lung cancer patients.

	Positive RKIP Expression (%)				Prognostic Value	Clinical Correlations	Molecule Analyzed (Technique)
	AC	SCC	AC+SCC	Nontumor			
Zhu C et al., 2012 [85]	-	47.7% (41/86)	47.7 % (41/86)	76.7 % (66/86)	-	LN metastasis; TMN stage; E-Cadherin expression	mRNA (RT-PCR)
Wang Q et al., 2014 [86]	49% (31/63)	32.7% (16/49)	41.9% (47/112)	Yes ^†^	-	LN metastasis; TMN stage	mRNA (QPCR)
Yan H et al., 2012 [87]	52.6% (30/57)	47.0% (48/102)	49.1% (78/159)	-	-	LN metastasis; TMN stage	Protein (IHC ^1^)
Shi-Yang X et al., 2017 [88]	53% (16/30)	79% (44/63)	64.5% (60/93)	-	Yes (independent)	LN metastasis; TMN stage; Radiotherapy resistance	Protein (IHC ^2^)
Wang A et al., 2017 [89]	-	-	51% (51/100)	Yes†	-	LN metastasis; TMN stage; Distant metastasis; phosphorylated STAT3	Protein (IHC ^3^ e WB)
Huerta-Yepez S et al., 2011 [90]	-	-	37.6% (140/372) ^4,^*	Yes†	Yes (independent) ^1^	LN metastasis; Age	Protein ^4^ (TMA, IHC, WB)

AC: adenocarcinoma; SCC: Squamous Cell Carcinoma; LN: Lymph Node; IHC: Immunohistochemistry; WB: Western Blot; TMA: Tissue Microarray; RT-PCR: semi-quantitative PCR: QPCR: Real Time PCR. ^†^ Comparison of expression between surrounding healthy tissue and tumor tissue was performed and found significantly lower in tumor tissues, but percentage of RKIP positivity in nontumor tissues were not discriminated. ^1^ RKIP antibody not specified. ^2^ Antibody: ab76582, Abcam, Cambridge, MA, USA (Dilution—1:400). ^3^ Antibody: reference 13006, Cell signaling Technology, Inc. (Danvers, MA, USA) (Dilution: 1:200). ^4^ Relative to phosphorylated RKIP: Rabbit-anti-human pRKIP from Santa Cruz Biotechnology (Dilution—1:250). * Calculated from the survival curves presented in the paper.

**Table 3:** Genomic alterations on PEBP1 gene in lung cancer (TCGA data) *.

Study Reference	Sample ID	Histology	PEBP1 Mutations	Mutation Type	PEBP1 CNA	Oncogenic Alterations
TCGA, Cell 2018 [113,114,115]	TCGA-05-4244-01	AC	G110W	Missense	no alteration	KRAS (G12C)
TCGA, Cell 2018 [113,114,115]	TCGA-97-7938-01	AC	L58V	Missense	no alteration	KRAS (G12C); ALK (E1299 *)
TCGA, Cell 2018 [113,114,115]	TCGA-34-5232-01	SCC	HECTD4-PEBP1	Fusion	no alteration	-
TCGA, Cell 2018 [113,114,115]	TCGA-66-2756-01	SCC	P71Rfs*22	FS del	no alteration	ROS1 (R1129S)
TCGA, Cell 2018 [113,114,115]	TCGA-66-2792-01	SCC	G57V	Missense	no alteration	EGFR AMP
Broad, Cell 2012 [118]	LUAD-B00416	AC	no alteration	-	AMP	-
TCGA, Provisional	TCGA-18-4083-01	SCC	no alteration	-	AMP	-
TCGA, Provisional	TCGA-44-7670-01	AC	no alteration	-	AMP	ALK (G446R)
TCGA, Nat Genet 2016 [119]	TCGA-50-5939-01	AC	no alteration	-	AMP	EGFR AMP
TCGA, Nat Genet 2016 [119]	LUAD-NYU994-Tumor	AC	no alteration	-	HOMDEL	-
TCGA, Provisional	TCGA-35-3615-01	AC	no alteration	-	HOMDEL	KRAS (G12C)
TCGA, Nature 2014 [120]	TCGA-55-6986-01	AC	no alteration	-	HOMDEL	ROS1 (Fusion)
TCGA, Nat Genet 2016 [119]	TCGA-75-6203-01	AC	no alteration	-	HOMDEL	-
TCGA, Cell 2018 [113,114,115]	TCGA-85-A513-01	SCC	no alteration	-	HOMDEL	-

www.cbioportal.org; CNA: copy number alterations; AC: adenocarcinoma; SCC: Squamous Cell Carcinoma; FS del: frameshift deletion; AMP: amplification; HOMDEL: homozygous deletion.

## Supplementary Materials

The following are available online at https://www.mdpi.com/2073-4409/8/5/442/s1, Figure S1: NSCLC TCGA data regarding PEBP1 copy number variations status according 12q chromosome status (refers to 510 AC cases and 484 SCC cases). All data is from TCGA PanCancer Atlas, available at www.cbioportal.org. NSCLC: Non-Small Cell Lung Carcinoma; AC: Adenocarcinoma; SCC: Squamous cell Carcinoma.
