# Supplementary material for: Current Status of Raf Kinase Inhibitor Protein (RKIP) in Lung Cancer: Behind RTK Signaling
Source: Cells. 2019 May 10;8(5):442. doi: 10.3390/cells8050442 (PMC6562953; doi:10.3390/cells8050442)
Supplement: Supplementary file 1 [file cells-08-00442-s001.pdf]

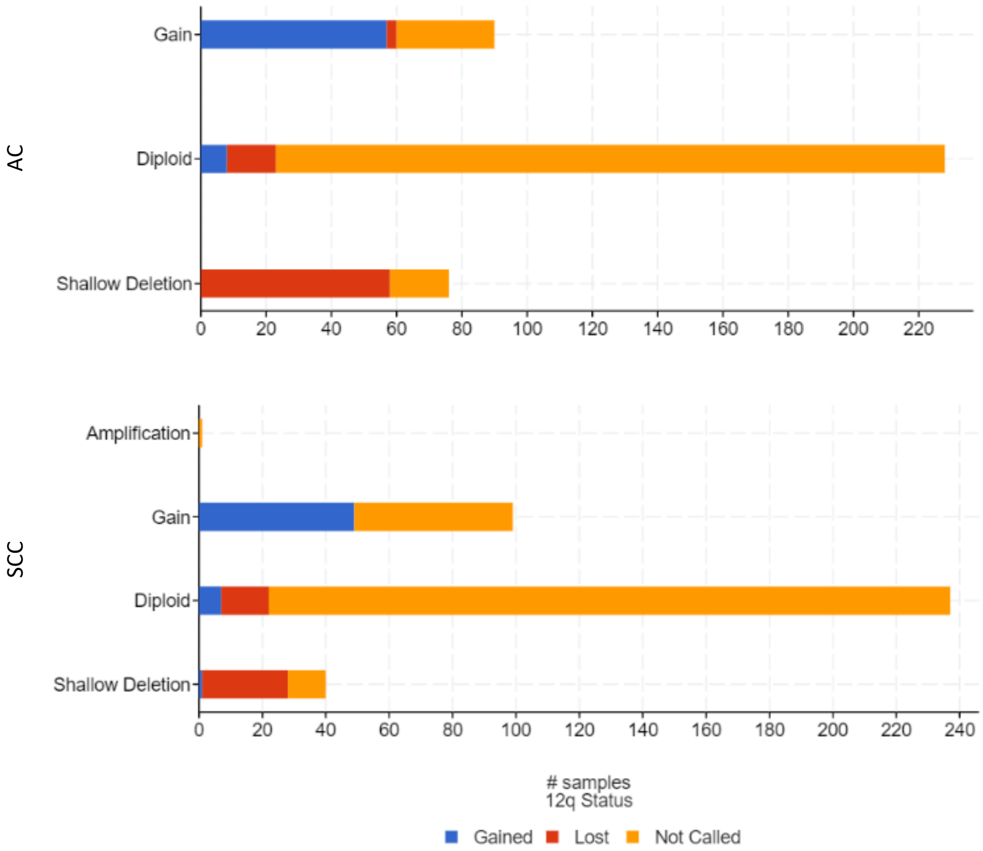

Figure S1: NSCLC TCGA data regarding *PEBP1* copy number variations status according 12q chromosome status (refers to 510 AC cases and 484 SCC cases). All the data is from TCGA PanCancer Atlas, available at www.cbioportal.org. NSCLC: Non-Small Cell Lung Carcinoma; AC: Adenocarcinoma; SCC: Squamous cell Carcinoma.
